# Hospital-Based Incidence Trends of Thyroid Cancer in Poland: Temporal Trends, Regional Disparities and Demographic Characteristics Based on a Nationwide Hospital Discharge Database (2012–2024)

**DOI:** 10.3390/cancers18193125

**Published:** 2026-09-27

**Authors:** Dorota Szydlarska, Katarzyna Lewtak, Marta Zuzanna Ciechomska, Anna Poznańska, Andrzej Śliwczyński, Krzysztof Kanecki

**Affiliations:** 1 Department of Digital Medicine, Implementation and Innovation, National Medical Institute of the Ministry of the Interior and Administration, Wołoska 137, 02-507 Warsaw, Poland; 2 Department of Family Medicine, Medical University of Warsaw, 02-091 Warsaw, Poland; 3 Department of Social Medicine and Public Health, Medical University of Warsaw, 02-091 Warsaw, Poland; 4 National Medical Institute of the Ministry of the Interior and Administration, Wołoska 137, 02-507 Warsaw, Poland; 5 Department of Population Health Monitoring and Analysis, National Institute of Public Health NIH—National Research Institute, 00-791 Warsaw, Poland; 6 Institute of Psychology and Human Sciences, WSEI University, 20-209 Lublin, Poland

**Keywords:** population-based database, thyroid cancer, hospital-based incidence, epidemiology, COVID-19

## Abstract

**Background**: Thyroid cancer (TC) is the most common endocrine malignancy, with a steadily increasing incidence observed worldwide. However, temporal trends and demographic patterns may vary across populations. **Objectives**: To assess hospital-based incidence rates, demographic characteristics, and selected epidemiological patterns of TC in Poland between January 2012 and December 2024. **Material and Methods**: This population-based, retrospective study utilized hospitalization records of TC patients in Poland obtained from the Nationwide General Hospital Morbidity Study. Only first-time hospitalizations in the study period with reported primary or secondary diagnoses of TC were included in the study. **Results**: The study cohort included 60,604 patients, and the mean annual hospital-based incidence rate was 12.2 per 100,000 inhabitants (95% CI: 11.0–13.4). The hospital-based incidence rate decreased from 15.2 in 2012 to 9.8 per 100,000 in 2020, followed by a marked increase, peaking to 16.6 per 100,000 in 2024. Women constituted 82.2% of cases, indicating a strong sex-related disparity. The mean age at diagnosis was 51.1 years (SD: 15.9). The mean age at diagnosis was 51.1 years (SD: 15.9), and a greater proportion of hospitalized thyroid cancer patients were urban than rural residents (67.4% vs. 32.6%). Marked regional variation was observed, with the highest rates recorded in selected northern and central voivodeships. **Conclusions**: TC hospitalization hospital-based incidence rates in Poland showed significant temporal variability, with the lowest rate in 2020, which may reflect disruptions in healthcare access during the COVID-19 pandemic and a possible diagnostic catch-up effect in subsequent years. Regional disparities may suggest the potential influence of environmental, socioeconomic, or healthcare-related factors.

## Simple Summary

Thyroid cancer is the most common cancer of the endocrine system, and its occurrence has increased worldwide over recent decades. However, long-term nationwide data describing trends in thyroid cancer in Poland remain limited. In this study, we analyzed hospital records of more than 60,000 patients with a first recorded hospitalization related to thyroid cancer between 2012 and 2024. We evaluated temporal trends, demographic characteristics, place of residence, and regional differences across the country. Women accounted for the vast majority of patients, and considerable variation was observed between different regions of Poland. Hospitalization rates decreased until 2020 and then increased markedly in subsequent years. These findings provide a comprehensive overview of thyroid cancer-related hospitalizations in Poland and may help improve understanding of disease patterns, healthcare utilization, and future public health planning.

## 1. Introduction

Thyroid cancer (TC) is the most prevalent malignancy of the endocrine system, comprising approximately 1–3% of all human cancers. It occurs two to four times more often in women than in men and ranks as the second most common cancer among individuals aged 0–49 years and the fifth most common among those aged 50–69 years [1]. Currently, TC is the seventh most commonly diagnosed malignancy worldwide, while its mortality rate ranks 24th among all cancers [2]. The global incidence of TC has risen markedly over the past three decades, largely driven by an increase in papillary thyroid carcinoma. Although many of these tumors are small and confined, epidemiological data indicate a growth in cases across all tumor sizes and stages, along with a rise in mortality specifically related to papillary TC. This pattern suggests that while enhanced detection and overdiagnosis play a major role, there is also evidence of a genuine increase in the occurrence of new TC cases [3].

Over the past 30 years, a consistent global rise in TC incidence has been observed [4]. Data from the European Network of Cancer Registries indicate that in 2012 the estimated incidence among women was approximately three times higher than among men, reaching 9.3 and 3.1 cases per 100,000 person-years, respectively [5]. Considerable international variation exists, with the highest incidence rates reported in Lithuania (15.5 per 100,000 person-years), followed by Italy (13.5), Austria (12.4), Croatia (11.4), and Luxembourg (11.1). In contrast, mortality remains low, estimated at 0.7 per 100,000 for women and 0.5 for men, and shows much less geographic and age variation [6].

Considering the histopathological diagnosis, the increase in TC incidence is attributable almost exclusively to a rise in diagnoses of differentiated TC, particularly papillary thyroid carcinoma. In comparison, the incidence of follicular, anaplastic, and medullary TC (MTC) has remained relatively unchanged over the past three decades. Papillary thyroid carcinoma is the most common form of TC in both adults and children. Since the 1980s, the criteria used to classify thyroid lesions as papillary thyroid carcinoma have evolved. Radiation exposure is a well-established risk factor for this malignancy [7]. Additional environmental factors include dietary iodine intake, obesity, hormonal influences, and environmental pollutants. Papillary thyroid carcinoma may also occur in familial settings, with approximately 5% of such cases associated with well-characterized germline driver mutations.

The growing use of advanced imaging modalities, biopsy techniques such as ultrasonography (USG) and fine-needle aspiration (FNA), and intensified medical surveillance, together with improved access to healthcare services, has substantially enhanced the detection of TC [8,9]. This phenomenon has led to widespread overdiagnosis, often accompanied by unnecessary treatment [10]. However, some authors contend that this trend cannot be explained solely by the overdiagnosis of microcarcinomas, since increases have also been observed in the incidence of larger tumors and more advanced-stage disease [11,12,13]. The U.S. Preventive Services Task Force has concluded that, in asymptomatic adults, the potential harms of TC screening outweigh its benefits [14]. Consequently, there is increasing support for more conservative, individualized management approaches, including active surveillance and risk-adapted treatment strategies [15]. TC remains the most prevalent endocrine malignancy worldwide, encompassing several distinct histological types—papillary, follicular, medullary, and anaplastic—each characterized by unique clinical and molecular features, with a rising incidence particularly among women [16].

Although TC incidence has been widely studied globally, data from Poland remain limited, particularly with regard to long-term, nationwide analyses. This study was therefore undertaken to address this gap and provide a comprehensive overview of temporal trends in Poland.

To our knowledge, this is the first nationwide study assessing thyroid cancer hospital-based incidence rates in Poland over a 13-year observation period (2012–2024).

The aim of this study was to evaluate temporal trends in TC hospital-based incidence rates in Poland in 2012–2024 using nationwide hospital discharge data, and to characterize differences according to age, sex, place of residence, and regional distribution, as well as the occurrence of selected comorbidities.

## 2. Methods

### 2.1. Data Source and Study Design

This retrospective, nationwide, population-based analysis was based on hospital discharge data for patients diagnosed with TC from 2012 to 2024, obtained from the Nationwide General Hospital Morbidity Study (NGHMS), conducted by the National Institute of Public Health NIH–National Research Institute within the framework of official statistics. The NGHMS collected anonymized data on all hospitalizations in Poland. This was a retrospective, non-interventional study based exclusively on anonymized secondary data. The study was conducted in accordance with the Declaration of Helsinki, and all methods complied with relevant guidelines and regulations. The study was approved by the Ethics Committee of the Medical University of Warsaw (approval number AKBE/220/2026).

### 2.2. Study Population

The study cohort comprised all hospitalization cases in Poland from 1 January 2012 to 31 December 2024 in which the ICD-10 code C73 (TC) was documented for the first occurrence in the study period. Eligibility criteria comprised: documentation of the ICD 10 code C73 as either a primary or secondary diagnosis, hospitalization occurring within the defined study period, and first-time appearance of the C73 code during the given hospital discharge. For hospital-based incidence calculations, only the initial TC-related hospitalization per patient within the study period was considered.

### 2.3. Variables and Definitions

For each hospital admission, information on patient age, sex, year of admission, and place of residence (urban or rural) was collected. The presence of selected comorbid conditions was determined using corresponding ICD-10 codes (hypothyroidism: E03 and nontoxic goiter: E04) documented in any diagnostic position.

Year-specific hospital-based incidence rates of TC were calculated by dividing the number of individuals with an initial hospitalization coded for TC in a given year by the total population of Poland for that year, with results reported per 100,000 population.

### 2.4. Statistical Analysis

Statistical analyses were performed using Statistica (TIBCO Software Inc., Palo Alto, CA, USA, version 13) and WINPEPI (developed by J.H. Abramson, Hebrew University, Jerusalem, Israel) [17,18]. For continuous variables, means, standard deviations, medians, and interquartile ranges were calculated. For categorical variables, counts and percentages were reported. Data from Statistics Poland on the annual population size were used as denominators for hospital-based incidence rate calculations [19]. Continuous variables were compared between groups using Student’s *t*-test, assuming approximate normality in large samples according to the central limit theorem [20]. Temporal trends in annual hospital-based incidence rates were analyzed using the Joinpoint Regression Program, Version 5.4.3 (Statistical Methodology and Applications Branch, Surveillance Research Program, National Cancer Institute, Bethesda, MD, USA). Log-linear models were fitted to annual incidence rates. The analysis allowed a maximum of one joinpoint because of the limited number of annual observations. Annual Percent Change (APC) and Average Annual Percent Change (AAPC), together with 95% confidence intervals (95% CI), were estimated using the Monte Carlo permutation method. Statistical significance was defined as *p* < 0.05.

## 3. Results

Between January 2012 and December 2024, a total of 149,337 hospitalizations due to TC were recorded nationwide. Among these, 60,604 patients were identified as having a first-time hospitalization with a diagnosis of TC, corresponding to a mean annual hospital-based incidence of 12.2 cases per 100,000 inhabitants.

Women accounted for the majority of cases (82.2%), reflecting the well-established female predominance of TC. The mean age of patients was 51.1 years, and 67.4% of cases were observed among urban residents (Table 1).

Figure 1 illustrates the annual number of first-time TC hospitalizations and corresponding incidence rates per 100,000 population between 2012 and 2024. A gradual decline in the hospital-based incidence rate was observed from 2012 until 2020, reaching the lowest value in 2020 (9.8 per 100,000). From 2021 onwards, a marked and sustained increase was noted, with the hospital-based incidence rate peaking in 2024 at 16.6 per 100,000 inhabitants. The hospital-based incidence rates observed in 2023–2024 were significantly higher than those recorded during the preceding decade, indicating a clear reversal of the earlier declining trend (*p* < 0.001).

Join point analysis identified a change in trend around 2020. Between 2012 and 2020, the hospital-based incidence rate decreased by an average of 2.7% per year (APC = −2.74%; 95% CI: −5.11 to −0.31). From 2020 to 2024, a marked increase was observed, with an APC of 13.11% annually (95% CI: 10.80–15.47; *p* = 0.001). For the entire study period, the average annual percent change (AAPC) was 1.28% (95% CI: −0.90 to 3.50) (Table 2).

Both sexes showed a decrease in hospital-based incidence around 2020 followed by an increase in subsequent years. However, formal trend analyses did not demonstrate statistically significant linear temporal trends within sex-specific series. Following the trend change observed around 2020, hospital-based incidence rates increased in both sexes, with a relatively stronger increase among men (APC = +15.75%) than among women (APC = +12.50%).

Among patients with a first TC-related hospitalization, women constituted 82.2% of cases (*n* = 49,794), while men accounted for 17.8% (*n* = 10,810). Throughout the entire study period, TC hospital-based incidence rate was consistently and substantially higher in women than in men. In both sexes, a declining trend in hospital-based incidence rate was observed between 2012 and 2020, followed by a pronounced increase thereafter. The post-2020 increase was relatively more pronounced among men than among women (APC: 15.75% vs. 12.50%) (Figure 2).

The 51. 1 ± 15.9 years and mean age among females were significantly younger than males (50.7, SD: 15.8 vs. 53.2, SD: 16.3; *p* < 0.001). Patient age ranged from 3 to 99 years, with an interquartile range of 39–64 years, indicating a predominance of middle-aged individuals. Over the study period, a gradual decrease in the mean age was observed, from 53.7 years in 2012 to approximately 49.8 years in 2024, with the lowest mean values observed between 2020 and 2022 and a subsequent stabilization thereafter. In male patients, a similar temporal pattern in age distribution was observed (Figure 3). The mean age showed a gradual decrease over time, declining from 54.7 in 2012 to 52.6 in 2024. The lowest mean age values in women and men were recorded between 2020 and 2022, followed by stabilization thereafter. In female patients, the mean age was 50.7 ± 15.8 years, with a median of 51 years and a wide age range (3–99 years) (Figure 3). A gradual decline in mean age was observed over time, decreasing from 53.5 years in 2012 to approximately 49.2 years in 2024. The lowest values were recorded between 2020 and 2022, with subsequent stabilization. The median age also decreased over time, indicating a shift toward younger age groups among female patients.

Notably, a trend toward a lower age at first hospitalization was observed in the years 2020–2024 compared with the period 2012–2019 in both sexes; however, the magnitude of this difference was greater among women.

Figure 4 presents the age distribution of thyroid cancer cases in males and females in 5-year age groups, together with the corresponding trend lines. The age distributions of thyroid cancer cases in women and men were broadly comparable, with the highest case frequencies observed in middle-aged and older adults. Women tended to reach their maximum number of cases at younger ages than men, although the overall shape of the age distributions remained similar between sexes (HD = 0.07).

Comparison of age-specific distributions showed a high degree of similarity between sexes (HD = 0.07), despite differences in the age groups with the highest observed numbers of cases.

Marked regional differences in hospital-based thyroid cancer incidence rates were observed across Polish voivodeships. To illustrate the most recent geographic distribution of the disease burden, regional rates for 2024 are presented in Figure 5. The highest hospitalization rates per 100,000 inhabitants were recorded in selected northern and central voivodeships.

Marked regional variation was observed across Polish voivodeships (Table 3). The largest number of cases was recorded in Mazowieckie (*n* = 9000), followed by Wielkopolskie (*n* = 7187) and Śląskie (*n* = 6568). When expressed relative to population size, the highest rates were observed in Świętokrzyskie (251.2 per 100,000), Podlaskie (234.2 per 100,000), and Zachodniopomorskie (224.2 per 100,000), whereas the lowest rates were found in Opolskie (53.0 per 100,000), Dolnośląskie (79.4 per 100,000), and Podkarpackie (94.4 per 100,000).

Among the analyzed comorbid conditions, hypothyroidism (ICD-10 code E03) and nontoxic goiter (ICD-10 code E04) were particularly prevalent. Figure 6 illustrates temporal trends in the prevalence of these comorbidities among patients with TC, showing persistently high frequencies throughout the study period. ICD-10 code E03 represents “Other hypothyroidism,” covering various forms of underactive thyroid not classified elsewhere, such as autoimmune (Hashimoto’s in hypothyroid phase), pharmacological, or idiopathic causes. It excludes congenital deficiency and iodine-deficiency hypothyroidism (E00–E02) and ICD-10 code E04 represents “Other Nontoxic Goiter,” covering thyroid gland enlargement (goiter) without hyperthyroidism or iodine deficiency. It indicates functional, non-autoimmune, and non-congenital swelling was present. Among the analyzed comorbidities, a significant increasing trend was observed for nontoxic goiter (ICD-10 code E04), with an average annual increase of 7.1% (*p* = 0.001).

Despite substantial differences in the absolute number of cases, temporal patterns were highly similar in women and men. This observation was supported by a strong correlation between the sex-specific time series (Pearson’s r = 0.95).

## 4. Discussion

In our study, covering the period 2012–2024, a marked variability in TC hospital-based incidence rate was observed, with an initial decline until 2020 followed by a significant and sustained increase in subsequent years, reaching a peak in 2024. The overall average annual percent change (AAPC) for the period 2012–2024 was 1.28%, indicating a slight increase in hospital-based thyroid cancer incidence rates over the entire study period despite the marked trend reversal observed around 2020. One possible explanation is reduced access to diagnostic services during the COVID-19 pandemic, followed by delayed case detection in subsequent years. The subsequent increase may therefore be attributed to intensified diagnostic activity and a catch-up effect related to previously delayed diagnoses within the healthcare system. However, the present study was not designed to directly evaluate the causes of this trend, and alternative explanations cannot be excluded.The observed rates should be interpreted as hospital-based incidence rates rather than true cancer incidence. The database captures first hospitalizations associated with TC and may therefore be influenced by healthcare accessibility, referral patterns, hospitalization practices, and diagnostic activity. Consequently, the results reflect both disease occurrence and healthcare system utilization. In contrast, in the United States, TC incidence increased between 2000 and 2013 and then declined significantly from 2014 to 2019 [21]. This reduction has been partly attributed to increased awareness of healthy lifestyle practices and changes in environmental exposures, including the well-established associations between TC, obesity, and environmental factors [22]. Importantly, U.S. data also demonstrated a significant increase in incidence-based mortality between 1994 and 2013, averaging approximately 1.1% per year overall and 2.9% per year among patients with advanced-stage papillary thyroid carcinoma (PTC), paralleling the rising incidence of advanced-stage disease since 1981 [23,24]. These observations challenge the assumption that the increase in PTC incidence can be explained solely by overdiagnosis [25,26]. The widespread use of ultrasonography, advanced imaging, and fine-needle aspiration biopsy has facilitated the detection of small, localized tumors—most of which follow an indolent course [27]. These diagnostic advancements likely explain the previously observed sharp rise in the incidence of small papillary TC [22,25,28]. Indeed, between 1992 and 2018, TC incidence increased by more than 200%, whereas mortality remained relatively stable over time [29]. This trend has been reported globally, particularly among younger individuals, although it is observed across all age groups [30]. Currently, TC is among the fastest-rising malignancies in the United States, ranking third in terms of growth rate, with an annual increase of approximately 3% over the past decade, resulting in a doubling of incidence over three decades [25,31,32,33]. Similar patterns have been reported in Canada, Australia, and Western Europe, although the underlying causes of this so-called “TC epidemic” remain incompletely understood [34,35,36,37,38]. Although the present analysis was based on first hospitalizations rather than cancer registry records, a substantial proportion of newly diagnosed cases is likely captured within the hospital database; however, the presented estimates should not be considered equivalent to true population-based incidence rates. In the Polish healthcare system, patients with newly suspected or diagnosed thyroid cancer are typically referred for further inpatient diagnostic evaluation and treatment, including radioiodine procedures and Thyrogen-stimulated assessments, which are predominantly performed in hospital settings. Consequently, a substantial proportion of newly diagnosed cases is likely to be captured within the hospital registry.

The age distribution of thyroid cancer cases was broadly similar in women and men, although women were diagnosed at a slightly younger mean age than men. In males, the highest number of hospitalizations was observed in the 55–70-year age group, whereas in females the distribution was more uniform across a broader age range of 35–70 years. This pattern may reflect underlying biological differences as well as sex-related variations in the onset and detection of thyroid diseases. In our study, the mean age at first hospitalization was approximately 51 years overall and was slightly higher in men than in women. However, a gradual decrease in the age at diagnosis was observed over the study period, particularly after 2020. These findings are consistent with international data. The temporal trends were generally consistent across both sexes. An interesting finding of the present study was the relatively greater increase in hospital-based incidence rates among men after 2020 compared with women (APC: +15.75% vs. +12.50%). Although TC remains markedly more common in women, this observation suggests that the recovery of diagnostic activity following the COVID-19 pandemic may have affected the sexes differently. Because the present analysis is based on first hospitalizations rather than cancer registry data, the observed trend may partly reflect differences in healthcare utilization and referral patterns. Further studies based on population-based cancer registries are needed to determine whether this phenomenon represents a true epidemiological change or is primarily related to healthcare system factors. Nevertheless, the trend reversal identified around 2020 was observed in both sexes, supporting the hypothesis that healthcare system–related factors contributed importantly to the observed epidemiological pattern. An analysis of cancer registries from 50 countries demonstrated that the age-standardized incidence of TC was 3.1 per 100,000 in men and 10.1 per 100,000 in women, with an increasing trend observed across most countries and all age groups, including individuals under 40 years of age, particularly in Korea, Poland, and China [30]. This increase was also reflected in countries with the highest growth rates, such as Korea (men: AAPC 25.3%, 95% CI 22.3–28.4, *p* < 0.001; women: AAPC 18.5%, 95% CI 16.2–20.9, *p* < 0.001) and China (men: AAPC 18.6%, 95% CI 12.1–25.5, *p* < 0.001; women: AAPC 13.3%, 95% CI 11.5–15.1, *p* < 0.001) [26]. Overall, these observations confirm a growing burden of TC in younger populations worldwide, emphasizing the need for targeted preventive strategies and careful consideration of the potential role of overdiagnosis.

In our study, the patterns of TC hospital-based incidence rate according to sex and geographic location were largely consistent with previously published findings [22,23,39]. However, data from the United States indicate that between 2014 and 2019 a significant decline in TC incidence occurred across various demographic and socioeconomic groups, as well as across tumor subtypes [21]. The most pronounced decreases in annual percent change (APC) for overall TC and papillary TC were observed among individuals aged 55–69 years, women, Black populations, those with higher socioeconomic status (SES), and urban residents. Additionally, declining hospital-based incidence of follicular thyroid carcinoma (FTC) was noted in women, Black populations, higher-SES groups, urban regions, and non-Hispanic populations. Further analyses reveal significant disparities in TC incidence across racial and socioeconomic groups. TC is observed approximately twice as often in white populations as in Black populations. Current incidence patterns indicate the highest rates among white individuals, followed by Asians and Pacific Islanders, American Indians/Alaska Natives, and the lowest rates among Black individuals [39]. Moreover, TC incidence is generally higher in non-Hispanic populations than in Hispanic populations, in both males and females. These findings highlight the role of socioeconomic factors, healthcare access, and potential environmental and genetic influences in shaping observed epidemiological patterns. According to GLOBOCAN data, TC-related mortality remains low, typically below 1 per 100,000 individuals in most countries for both sexes. South Korea has been identified as having the highest incidence-to-mortality ratio among both men and women [40]. Importantly, no substantial differences in mortality rates have been observed between countries with high and low or medium Human Development Index (HDI). The highest incidence rates are reported in South Korea for both sexes, while in regions such as East Asia, Australia/New Zealand, North America, Oceania, and Pacific Island countries, women are affected more frequently than men [41]. Overall, TC is considerably more common in women worldwide, with incidence rates approximately 2 to 4 times higher than in men [42].

An additional finding was the significant increase in the prevalence of nontoxic goiter (E04) over time. This observation may reflect increased diagnostic activity, changes in coding practices, or a growing burden of concomitant benign thyroid disease among patients diagnosed with thyroid cancer.

The present study demonstrated that a greater proportion of hospitalized thyroid cancer patients were urban residents compared to rural residents. In Poland, substantial disparities in access to healthcare services exist between these areas, largely due to the uneven distribution of medical personnel and healthcare infrastructure [43]. Individuals living in rural areas more frequently experience limited access to physicians, particularly specialists, as well as longer waiting times for medical services, which may negatively affect the diagnostic process and treatment outcomes [44]. This difference may therefore reflect improved access to diagnostic services in urban settings rather than a true increase in disease risk, although greater exposure to environmental pollution in urban agglomerations should also be considered as a potential contributing factor [45]. Consistent with this interpretation, the study by Morris et al. demonstrated that better access to healthcare—reflected by both sociodemographic and age-related factors—was associated with higher detection rates of papillary TC [46]. Despite a marked increase in incidence over three decades, mortality remained stable, supporting the role of overdiagnosis driven by the detection of subclinical disease. Environmental pollutants have been associated with a wide range of adverse health outcomes, including metabolic disorders, allergies, reproductive disorders, and malignancies, including TC [47,48,49,50,51,52]. Overall, our findings support the hypothesis that access to healthcare plays a key role in shaping observed hospital-based incidence rate patterns of TC.

Marked regional differences in TC hospitalization rates were observed across Polish voivodeships in 2024, with the highest values recorded in selected northern and central voivodeships. In Poland, patients may receive specialist treatment in any region of the country regardless of their place of residence. However, the regional analyses performed in this study were based on the recorded place of residence of each patient, rather than the location of the treating hospital. Therefore, the observed regional differences are unlikely to be explained by interregional referral patterns or concentration of treatment in highly specialized centers.

In Poland, significant disparities in disposable household income have been reported, with the highest levels observed in the Mazowieckie, Wielkopolskie, and Pomorskie voivodeships and the lowest in eastern regions. Notably, some areas with the highest hospitalization rates are among the least economically developed regions, which may also reflect lower health awareness and differences in healthcare utilization. Globally, the incidence of TC continues to rise and currently ranks among the most common malignancies. According to GLOBOCAN data, approximately 586,000 new cases were recorded worldwide in 2020, placing TC tenth globally, with the majority occurring in countries with high or very high Human Development Index (HDI) [40]. The Western Pacific region accounts for nearly half of all cases despite representing only one-quarter of the global population. Projections indicate that by 2040, incidence will increase by nearly 30% and mortality by 67%, with the greatest increases expected in Africa. These findings highlight marked global disparities, with higher incidence observed in highly developed regions and disproportionately higher mortality in low-HDI countries, likely due to differences in access to healthcare and environmental exposures [53].

Exposure to ionizing radiation, particularly during childhood, remains the most well-established risk factor for papillary TC. The Chernobyl nuclear disaster in 1986 resulted in substantial environmental contamination and high thyroid radiation doses in affected regions of Eastern Europe, including parts of Poland [54]. The northeastern regions were particularly affected by radioactive fallout. The pathogenesis of TC is complex and multifactorial, involving genetic, hormonal, and environmental influences. In our study, hypothyroidism and nontoxic goiter were among the most frequently recorded coexisting conditions in patients hospitalized with thyroid cancer. However, because of the cross-sectional nature of the available data, it was not possible to determine whether these disorders preceded thyroid cancer diagnosis or were identified during the diagnostic work-up. Therefore, no conclusions regarding causal relationships can be drawn. Similar findings have been reported in population-based studies, where benign thyroid diseases and radiation exposure were associated with increased risk [55].

Overall, the observed trends in TC hospital-based incidence rate in Poland may reflect a complex interplay between improved diagnostic intensity, healthcare system factors, and potential environmental and lifestyle influences. While increased detection and overdiagnosis likely contribute substantially to the rising incidence, the consistency of trends across demographic groups and the persistence of geographic variations suggest that a true increase in disease occurrence cannot be entirely excluded.

## 5. Limitations of the Study

This study has several limitations that should be considered when interpreting the results. First, the analysis was based on administrative hospital discharge data, which may be subject to coding errors and misclassification of diagnoses. The use of ICD-10 codes does not allow for detailed clinical verification of cases or differentiation between histopathological subtypes of TC, which may influence the observed epidemiological patterns. Second, the study included only hospitalized patients with a first recorded diagnosis of TC. Consequently, cases diagnosed and managed entirely in outpatient settings may not have been captured, potentially leading to an underestimation of the true incidence. Furthermore, because information prior to 2012 was unavailable, some patients with thyroid cancer diagnosed before the study period may have been misclassified as incident cases during the first years of observation. Therefore, a potential left-censoring effect, particularly in 2012, cannot be excluded.

Third, the retrospective observational design precludes establishing causal relationships between the identified factors (e.g., age, sex, place of residence) and TC occurrence. Furthermore, incidence rates were not age-standardized because age-specific population denominators were not available for all analyses. Consequently, the observed differences between sexes, calendar years, and regions may partially reflect differences in underlying population age structures rather than true differences in thyroid cancer occurrence. Therefore, these findings should be interpreted cautiously.

Finally, the database lacked detailed information on potential confounding variables, such as individual socioeconomic status, environmental exposures, iodine intake, lifestyle factors (e.g., obesity, smoking), and genetic predisposition, which may have influenced the results. Furthermore, incidence rates were not age-standardized; therefore, changes in population age structure over time may have influenced the observed trends.

## 6. Conclusions

The present study demonstrated significant temporal variability in TC hospital-based incidence rate in Poland between 2012 and 2024, with a decline observed until 2020 followed by a marked increase in subsequent years. This pattern may be associated with disruptions in healthcare access during the COVID-19 pandemic and subsequent recovery of diagnostic activity.

A clear predominance of TC cases among women was confirmed, along with a consistent trend of higher hospital-based incidence rate across the entire study period. The mean age at first hospitalization was approximately 51 years in the overall cohort and was slightly higher among men than women. A gradual decrease in age at diagnosis was observed, particularly in recent years, indicating a shift toward younger age groups. A greater proportion of hospitalized thyroid cancer patients were urban residents (67.4%). Further research is warranted to comprehensively assess the impact of socioeconomic factors, access to healthcare services, and environmental exposures on the observed health outcomes.

Finally, hypothyroidism and nontoxic goiter were among the most commonly recorded coexisting conditions. These findings describe patterns of coexistence and should not be interpreted as evidence of a causal relationship with thyroid cancer.

## Figures and Tables

**Figure 1:**
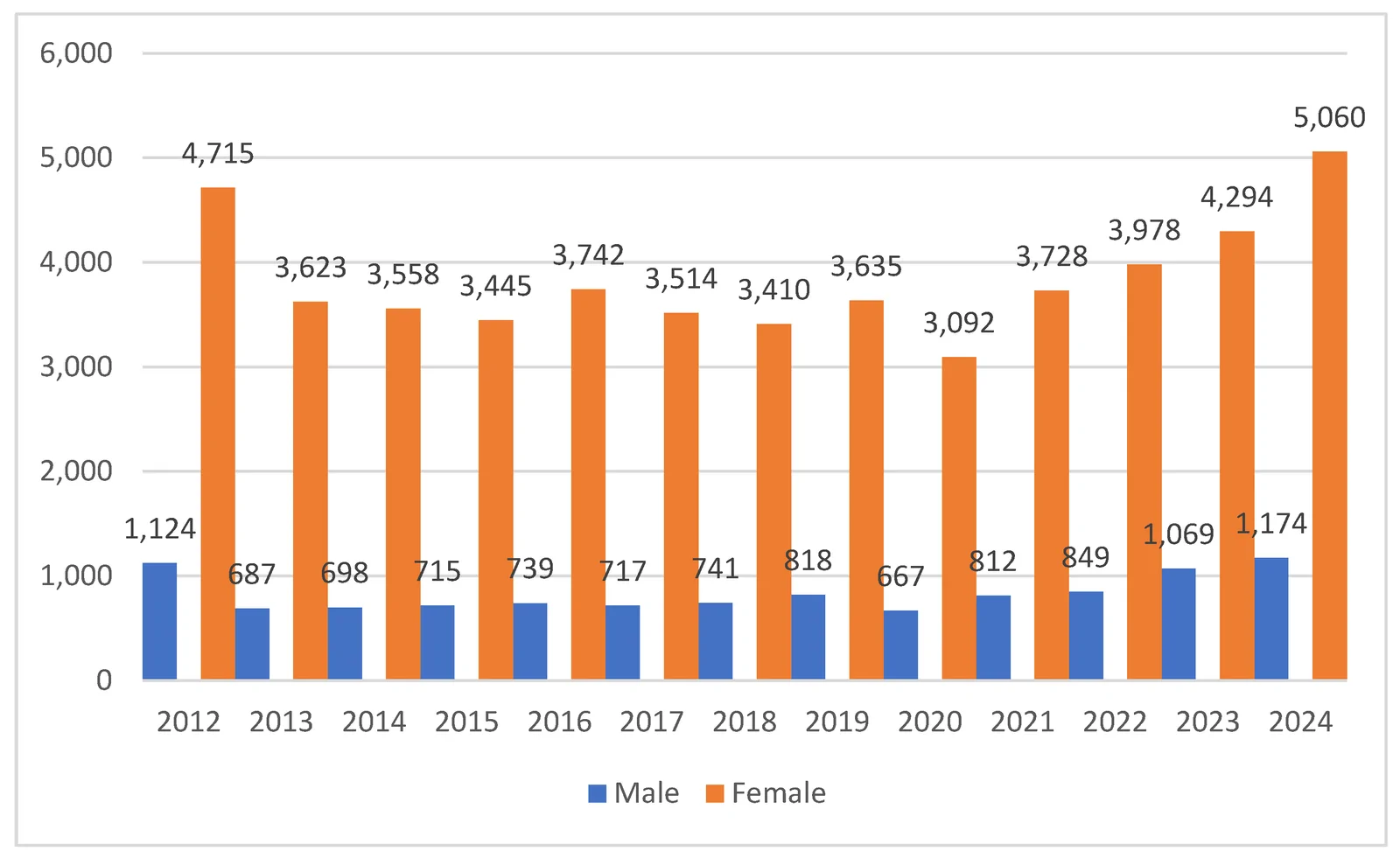
Distribution of number of patients by sex and time between 2012 and 2024.

**Figure 2:**
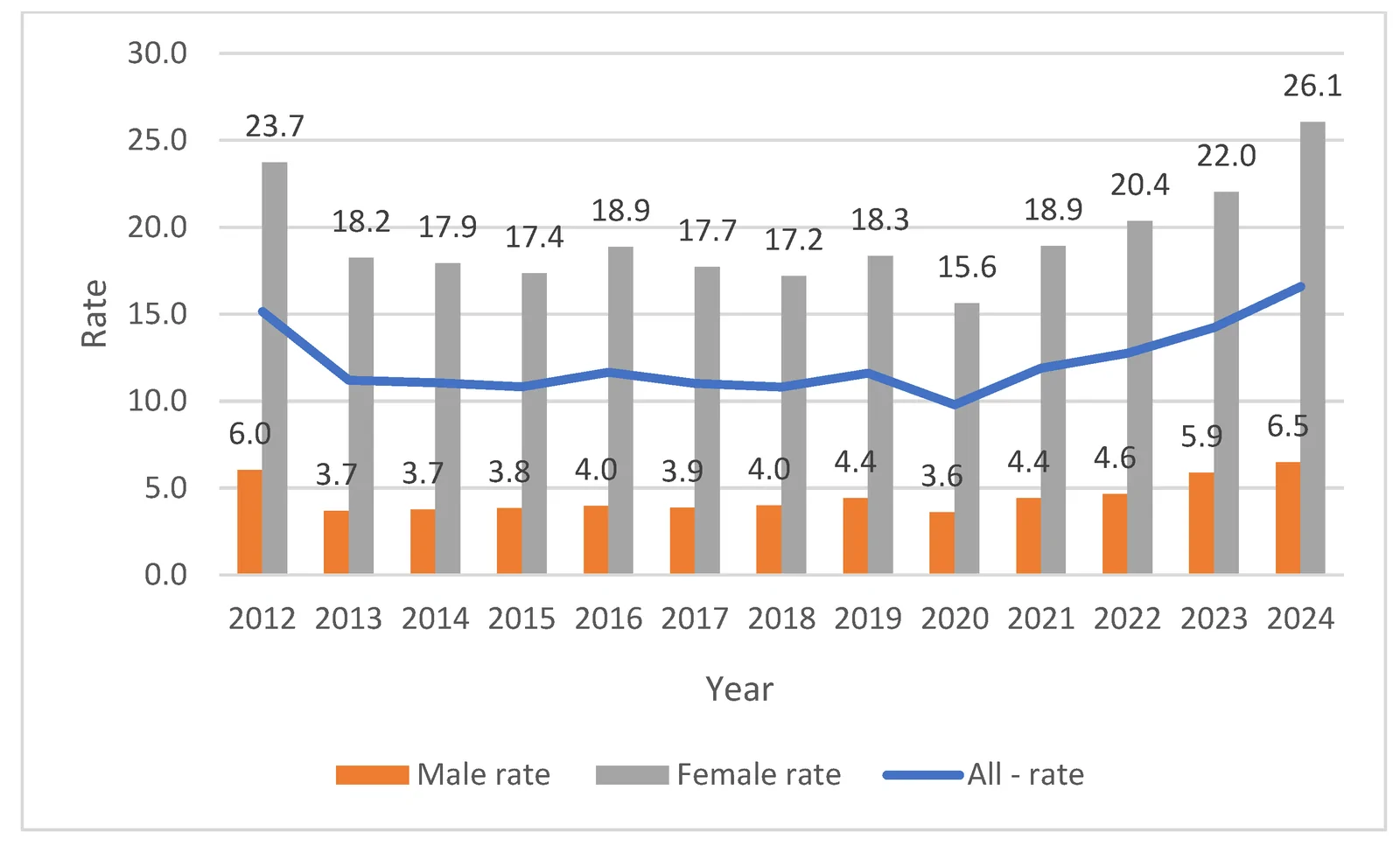
Sex-specific trends in thyroid cancer hospital-based incidence rates, 2012–2024.

**Figure 3:**
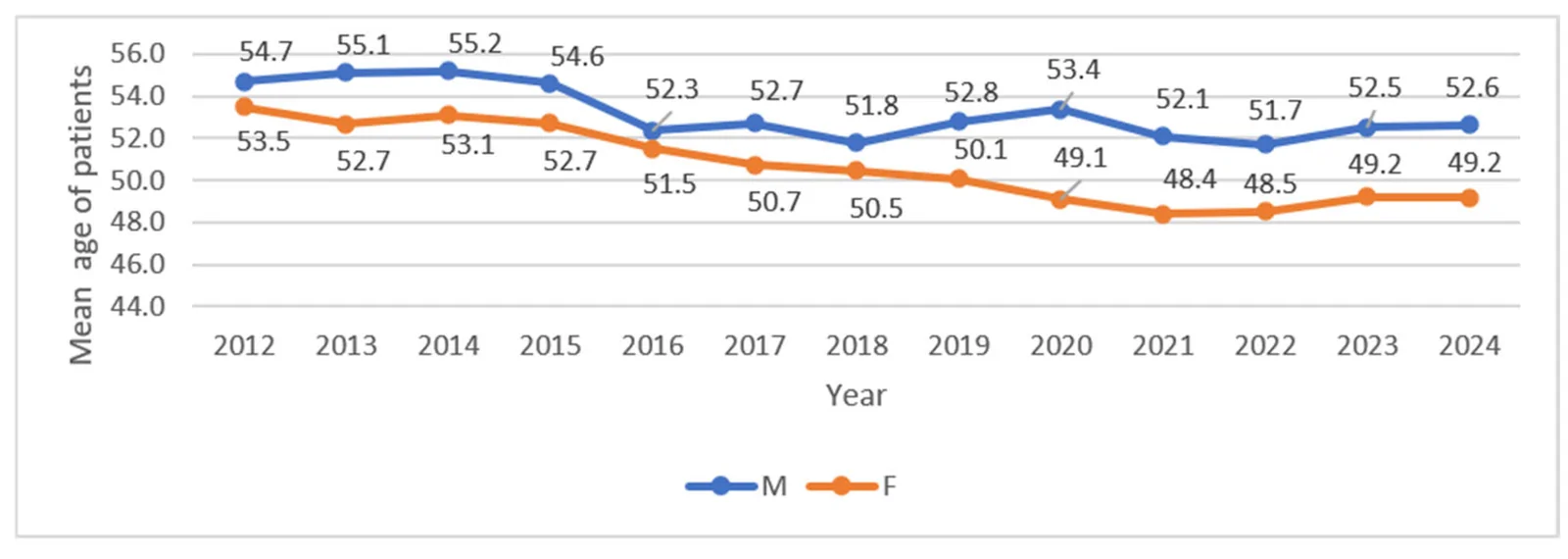
Mean age at first hospitalization for thyroid cancer by sex.

**Figure 4:**
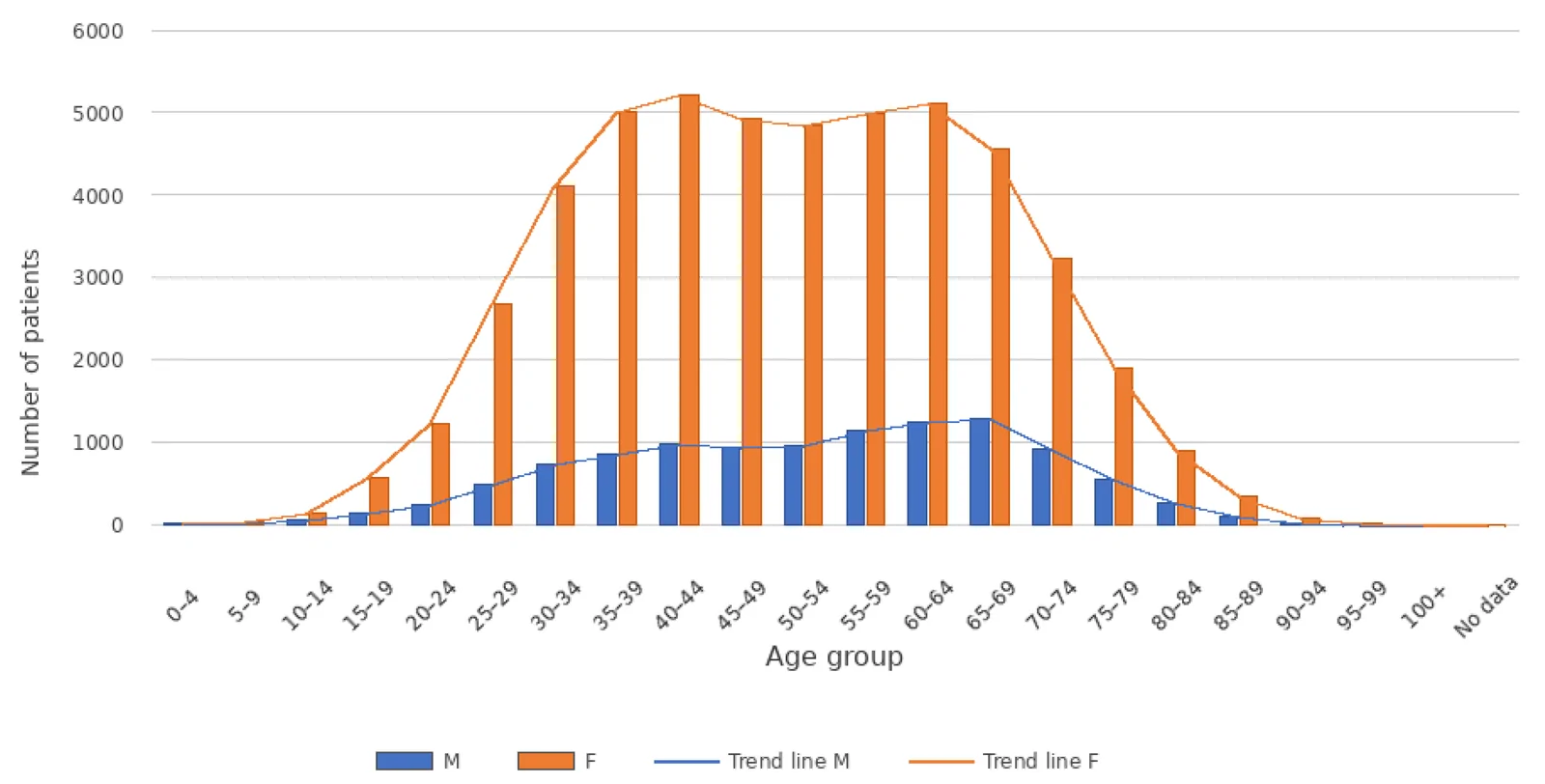
Distribution of the number of hospitalization cases in males and females by age in 5-year intervals.

**Figure 5:**
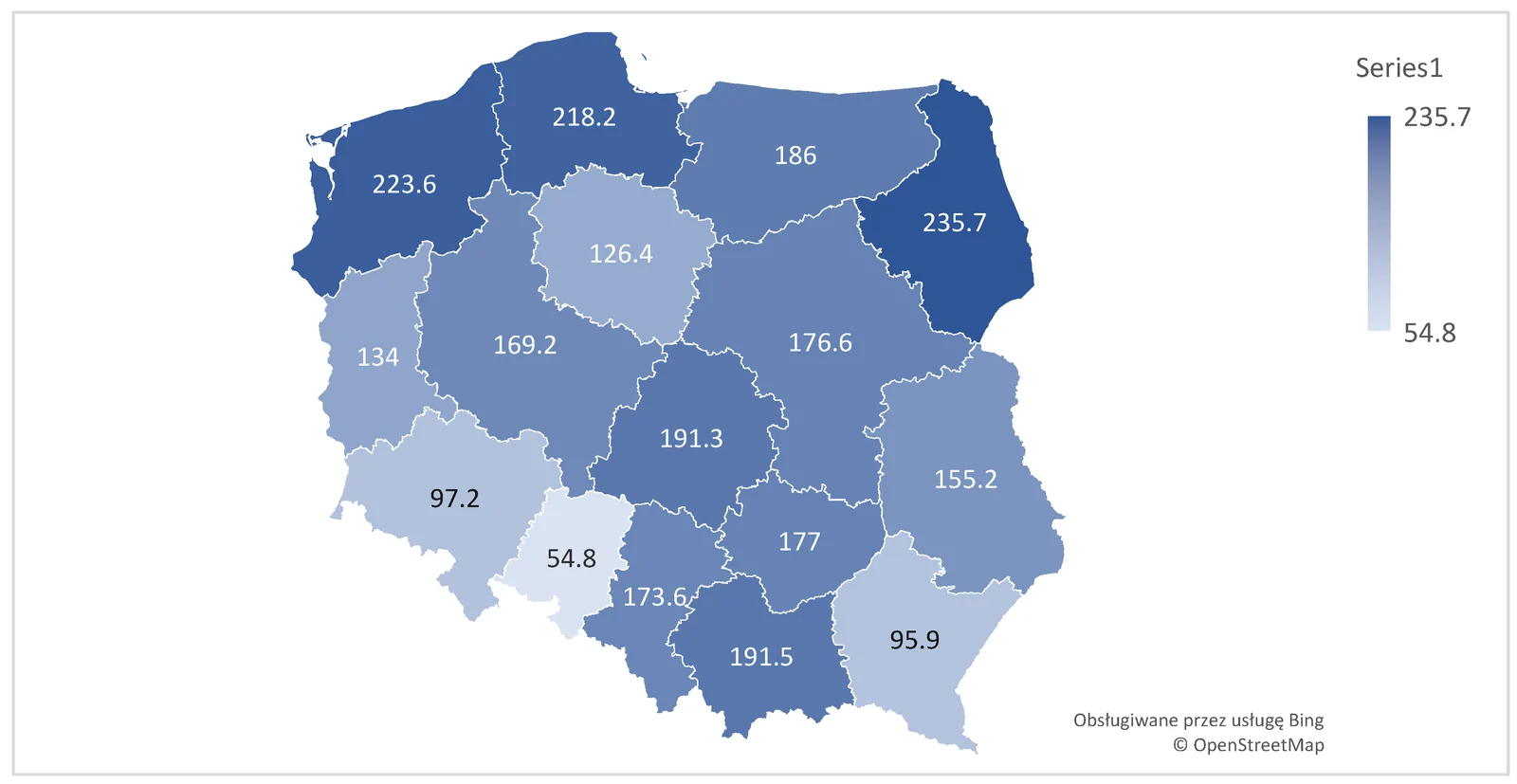
Regional variation in hospital-based thyroid cancer incidence rates per 100,000 inhabitants across Polish voivodeships in 2024.

**Figure 6:**
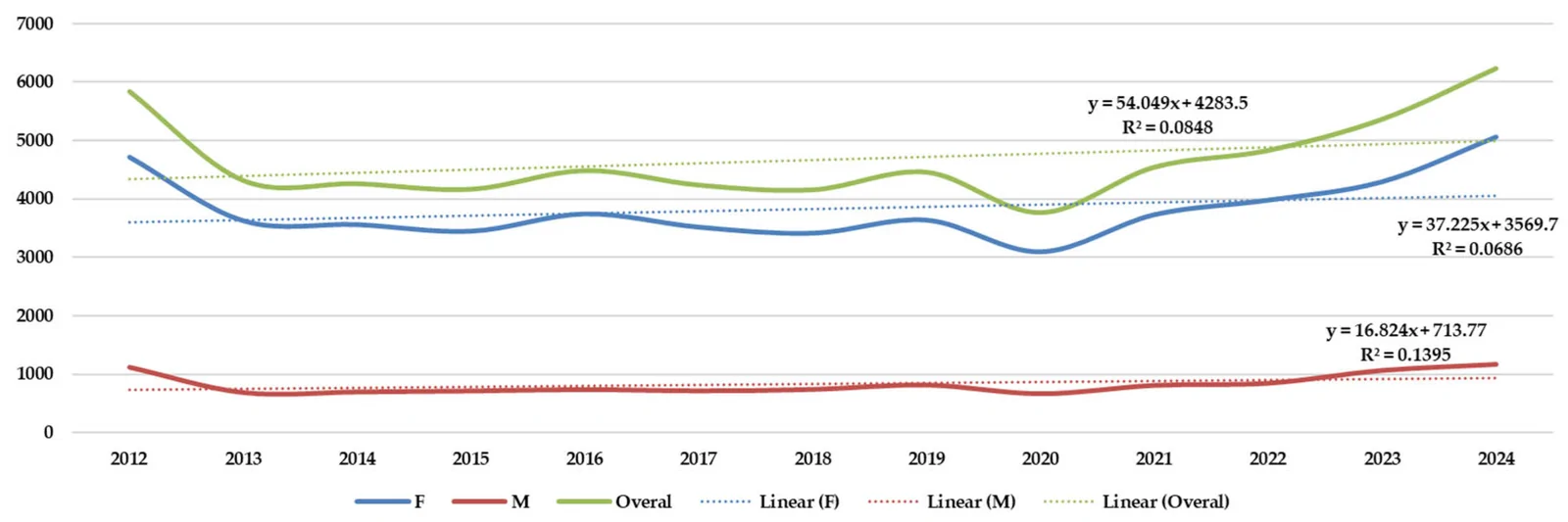
Temporal trends in hospitalizations for men, women and total population.

**Table 1:** Baseline characteristics of patients hospitalized for thyroid cancer in Poland, 2012–2024.

Characteristic	Value
Number of patients	60,604
Age, years, mean ± SD	51.1 ± 15.9
Age, median (IQR)	51 (39–64)
Age range, years	3–99
Female sex, *n* (%)	49,794 (82.2)
Male sex, *n* (%)	10,810 (17.8)
Female-to-male ratio	4.6:1
Urban residence, *n* (%)	40,871 (67.4)
Rural residence, *n* (%)	19,728 (32.6)
Other/unknown residence, *n* (%)	5 (<0.1)

Data are presented as *n* (%) unless otherwise indicated. IQR, interquartile range; SD, standard deviation.

**Table 2:** Relative percentage changes in hospital-based thyroid cancer incidence rates during 2012–2020, 2020–2024, and the entire study period (2012–2024).

Population	% Change 2012–2020	% Change 2020–2024	% Change 2012–2024
Total population	−35.62	+65.84	+0.55
Men	−40.66	+76.01	+0.36
Women	−34.42	+63.65	+0.59

**Table 3:** Cumulative hospitalization rate during 2012–2024.

Voivodeship	Cases (*n*)	% of All Cases
Mazowieckie	9000	14.85%
Wielkopolskie	7187	11.86%
Śląskie	6568	10.84%
Małopolskie	5815	9.60%
Pomorskie	4996	8.24%
Łódzkie	4699	7.75%
Zachodniopomorskie	3647	6.02%
Świętokrzyskie	2922	4.82%
Podlaskie	2659	4.39%
Warmińsko-mazurskie	2510	4.14%
Dolnosląskie	2283	3.77%
Kujawsko-pomorskie	2268	3.74%
Lubelskie	2147	3.54%
Podkarpackie	1952	3.22%
Lubuskie	1451	2.39%

Number of hospitalization cases recorded during 2012–2024 relative to the voivodeship population in 2024.

## Data Availability

The original contributions presented in this study are included in the article. Further inquiries can be directed to the corresponding author.

## References

[1] Bernier M., Withrow D.R., de Gonzalez A.B., Lam C.J.K., Linet M.S., Kitahara C.M., Shiels M.S. (2019). Trends in pediatric thyroid cancer incidence in the United States, 1998–2013. Cancer.

[2] Benedetti M., Zona A., Contiero P., D’armiento E., Iavarone I., AIRTUM Working Group (2020). Incidence of Thyroid Cancer in Italian Contaminated Sites. Int. J. Environ. Res. Public Health.

[3] Seib C.D., Sosa J.A. (2019). Evolving Understanding of the Epidemiology of Thyroid Cancer. Endocrinol. Metab. Clin. N. Am..

[4] Filetti S., Durante C., Hartl D., Leboulleux S., Locati L., Newbold K., Papotti M., Berruti A. (2019). Thyroid cancer: ESMO Clinical Practice Guidelines for diagnosis, treatment and follow-up. Ann. Oncol..

[5] (2019). European Network of Cancer Registries Factsheets. https://www.encr.eu/sites/default/files/factsheets/ENCR_Factsheet_Thyroid_2017-2.pdf.

[6] Maso L.D., Tavilla A., Pacini F., Serraino D., van Dijk B., Chirlaque M., Capocaccia R., Larrañaga N., Colonna M., Agius D. (2017). Survival of 86,690 patients with thyroid cancer: A population-based study in 29 European countries from EUROCARE-5. Eur. J. Cancer.

[7] Lam A.K. (2022). Papillary Thyroid Carcinoma: Current Position in Epidemiology, Genomics, and Classification. Methods Mol. Biol..

[8] James B.C., Mitchell J.M., Jeon H.D., Vasilottos N., Grogan R.H., Aschebrook-Kilfoy B. (2018). An update in international trends in incidence rates of thyroid cancer, 1973–2007. Cancer Causes Control..

[9] Li M., Maso L.D., Vaccarella S. (2020). Global trends in thyroid cancer incidence and the impact of overdiagnosis. Lancet Diabetes Endocrinol..

[10] Ahn H.S., Welch H.G. (2015). South Korea’s Thyroid-Cancer “Epidemic”—Turning the Tide. New Engl. J. Med..

[11] Malaguarnera R., Vella V., Pellegriti G., Belfiore A. (2018). Editorial: Clinical and Molecular Epidemiology of Thyroid Cancer of Follicular Origin. Front. Endocrinol..

[12] Sanabria A., Kowalski L.P., Shah J.P., Nixon I.J., Angelos P., Williams M.D., Rinaldo A., Ferlito A. (2017). Growing incidence of thyroid carcinoma in recent years: Factors underlying overdiagnosis. Head Neck.

[13] Paulson V.A., Rudzinski E.R., Hawkins D.S. (2019). Thyroid Cancer in the Pediatric Population. Genes.

[14] Bibbins-Domingo K., Grossman D.C., Curry S.J. (2017). Screening for thyroid cancer: US Preventive Services Task Force Recommendation Statement. JAMA.

[15] Ringel M.D., Sosa J.A., Baloch Z., Bischoff L., Bloom G., Brent G.A., Brock P.L., Chou R., Flavell R.R., Goldner W. (2025). 2025 American Thyroid Association Management Guidelines for Adult Patients with Differentiated Thyroid Cancer. Thyroid.

[16] Forma A., Kłodnicka K., Pająk W., Flieger J., Teresińska B., Januszewski J., Baj J. (2025). Thyroid Cancer: Epidemiology, Classification, Risk Factors, Diagnostic and Prognostic Markers, and Current Treatment Strategies. Int. J. Mol. Sci..

[17] TIBCO Software Inc. Statistica (Data Analysis Software System), Version 13. https://www.statsoft.pl/programy/statistica/.

[18] Abramson J.H. (2011). WINPEPI updated: Computer programs for epidemiologists, and their teaching potential. Epidemiol. Perspect. Innov..

[19] Statistics Poland. https://stat.gov.pl.

[20] Lumley T., Diehr P., Emerson S., Chen L. (2002). The importance of the normality assumption in large public health data sets. Annu. Rev. Public Health.

[21] Yu J. (2023). Trends in the incidence of thyroid cancer among US persons from 2000 to 2019. Eur. J. Cancer Prev..

[22] Enewold L., Zhu K., Ron E., Marrogi A.J., Stojadinovic A., Peoples G.E., Devesa S.S. (2009). Rising Thyroid Cancer Incidence in the United States by Demographic and Tumor Characteristics, 1980–2005. Cancer Epidemiol. Biomark. Prev..

[23] Lim H., Devesa S.S., Sosa J.A., Check D., Kitahara C.M. (2017). Trends in Thyroid Cancer Incidence and Mortality in the United States, 1974–2013. J. Am. Med. Assoc..

[24] Howlader N., Noone A.M., Krapcho M., Miller D., Bishop K., Altekruse S.F., Kosary C.L., Yu M., Ruhl J., Tatalovich Z. (2016). SEER Cancer Statistics Review, 1975–2013.

[25] Davies L., Welch H.G. (2014). Current Thyroid Cancer Trends in the United States. JAMA Otolaryngol. Head Neck Surg..

[26] Morris L.G., Tuttle R.M., Davies L. (2016). Changing Trends in the Incidence of Thyroid Cancer in the United States. JAMA Otolaryngol. Head Neck Surg..

[27] Sosa J.A., Hanna J.W., Robinson K.A., Lanman R.B. (2013). Increases in thyroid nodule fine-needle aspirations, operations, and diagnoses of thyroid cancer in the United States. Surgery.

[28] Mao Y., Xing M. (2016). Recent incidences and differential trends of thyroid cancer in the United States. Endocr. Relat. Cancer.

[29] Alagoz O., Zhang Y., Arroyo N., Fernandes-Taylor S., Yang D.-Y., Krebsbach C., Venkatesh M., Hsiao V., Davies L., Francis D.O. (2023). Modeling Thyroid Cancer Epidemiology in the United States Using Papillary Thyroid Carcinoma Microsimulation Model. Value Health.

[30] Huang J., Ngai C.H., Deng Y., Pun C.N., Lok V., Zhang L., Xu Q., Lucero-Prisno D.E., Xu W., Zheng Z.-J. (2023). Incidence and mortality of thyroid cancer in 50 countries: A joinpoint regression analysis of global trends. Endocrine.

[31] Davies L., Ouellette M., Hunter M., Welch H.G. (2010). The increasing incidence of small thyroid cancers: Where are the cases coming from?. Laryngoscope.

[32] Morris L.G., Myssiorek D. (2010). Improved detection does not fully explain the rising incidence of well-differentiated thyroid cancer: A population-based analysis. Am. J. Surg..

[33] Morris L.G.T., Sikora A.G., Myssiorek D., De Lacure M.D. (2008). The Basis of Racial Differences in the Incidence of Thyroid Cancer. Ann. Surg. Oncol..

[34] Burgess J.R. (2002). Temporal trends for thyroid carcinoma in Australia: An increasing incidence of papillary thyroid carcinoma (1982–1997). Thyroid.

[35] Colonna M., Grosclaude P., Remontet L., Schvartz C., Mace-Lesech J., Velten M., Guizard A., Tretarre B., Buemi A.V., Arveux P. (2002). Incidence of thyroid cancer in adults recorded by French cancer registries (1978–1997). Eur. J. Cancer.

[36] Kilfoy B.A., Zheng T., Holford T.R., Han X., Ward M.H., Sjodin A., Zhang Y., Bai Y., Zhu C., Guo G.L. (2009). International patterns and trends in thyroid cancer incidence, 1973–2002. Cancer Causes Control..

[37] Liu S., Semenciw R., Ugnat A.-M., Mao Y. (2001). Increasing thyroid cancer incidence in Canada, 1970–1996: Time trends and age-period-cohort effects. Br. J. Cancer.

[38] Leenhardt L., Grosclaude P., Chérié-Challine L. (2004). Increased Incidence of Thyroid Carcinoma in France: A True Epidemic or Thyroid Nodule Management Effects? Report from the French Thyroid Cancer Committee. Thyroid.

[39] Magreni A., Bann D.V., Schubart J.R., Goldenberg D. (2015). The Effects of Race and Ethnicity on Thyroid Cancer Incidence. Arch. Otolaryngol. Neck Surg..

[40] Pizzato M., Li M., Vignat J., Laversanne M., Singh D., La Vecchia C., Vaccarella S. (2022). The epidemiological landscape of thyroid cancer worldwide: GLOBOCAN estimates for incidence and mortality rates in 2020. Lancet Diabetes Endocrinol..

[41] Bray F., Ferlay J., Soerjomataram I., Siegel R.L., Torre L.A., Jemal A. (2018). Global cancer statistics 2018: GLOBOCAN estimates of incidence and mortality worldwide for 36 cancers in 185 countries. CA A Cancer J. Clin..

[42] Sung H., Ferlay J., Siegel R.L., Laversanne M., Soerjomataram I., Jemal A., Bray F. (2021). Global Cancer Statistics 2020: GLOBOCAN Estimates of Incidence and Mortality Worldwide for 36 Cancers in 185 Countries. CA A Cancer J. Clin..

[43] Sowada C., Sagan A., Kowalska-Bobko I., Badora-Musial K., Bochenek T., Domagala A., Dubas-Jakobczyk K., Kocot E., Mrozek-Gasiorowska M., Sitko S. (2019). Poland: Health System Review. Health Syst. Transit..

[44] Lechowski Ł., Jasion A. (2021). Spatial Accessibility of Primary Health Care in Rural Areas in Poland. Int. J. Environ. Res. Public Health.

[45] Rój J. (2026). Geographical Inequalities in the Distribution of Healthcare Specialists in Poland. Healthcare.

[46] Morris L.G., Sikora A.G., Tosteson T.D., Davies L. (2013). The Increasing Incidence of Thyroid Cancer: The Influence of Access to Care. Thyroid.

[47] Le Magueresse-Battistoni B., Vidal H., Naville D. (2018). Environmental Pollutants and Metabolic Disorders: The Multi-Exposure Scenario of Life. Front. Endocrinol..

[48] Ring J., Eberlein-Koenig B., Behrendt H. (2001). Environmental pollution and allergy. Ann. Allergy Asthma Immunol..

[49] Conti G.O., Calogero A.E., Giacone F., Fiore M., Barchitta M., Agodi A., Ferrante M. (2017). B(a)P adduct levels and fertility: A cross-sectional study in a Sicilian population. Mol. Med. Rep..

[50] Cohen A.J., Pope C.A. (1995). Lung cancer and air pollution. Environ. Health Perspect..

[51] Roman S.A. (2003). Endocrine tumors: Evaluation of the thyroid nodule. Curr. Opin. Oncol..

[52] Marcello M.A., Malandrino P., Almeida J.F.M., Martins M.B., Cunha L.L., Bufalo N.E., Pellegriti G., Ward L.S. (2014). The influence of the environment on the development of thyroid tumors: A new appraisal. Endocr.-Relat. Cancer.

[53] Shank J.B., Are C., Wenos C.D. (2021). Thyroid Cancer: Global Burden and Trends. Indian J. Surg. Oncol..

[54] Krajewski P. (2002). Rekonstrukcja dawek na tarczycę dla ludności Polski po awarii elektrowni w Czarnobylu (weryfikacja badań modelowych). Endokrynol. Pol..

[55] Parad M.T., Fararouei M., Mirahmadizadeh A.R., Afrashteh S. (2021). Thyroid cancer and its associated factors: A population-based case-control study. Int. J. Cancer.

